# 1700 nm dispersion managed mode-locked bismuth fiber laser

**DOI:** 10.1038/srep24876

**Published:** 2016-04-21

**Authors:** Teppo Noronen, Sergei Firstov, Evgeny Dianov, Oleg G. Okhotnikov

**Affiliations:** 1 Optoelectronics Research Centre, Tampere University of Technology, Korkeakoulunkatu 3, 33720 Tampere, Finland; 2 Fiber Optics Research Center, Russian Academy of Sciences, 38 Vavilov Street, 119333 Moscow, Russia; 3 Ulyanovsk State University, L. Tolstoy str. 42, 432017 Ulyanovsk, Russia

## Abstract

We demonstrate the first 1.7 μm bismuth-doped fiber laser generating ultrashort pulses via passive mode-locking. Pulse operation has been achieved for both anomalous and normal dispersion of the laser cavity owing to broadband characteristics of carbon nanotube saturable absorber. The laser delivered 1.65 ps pulses in net anomalous dispersion regime. In normal dispersion regime, the laser delivered 14 ps pulses which could be compressed to 1.2 ps using external fiber compressor.

Bismuth-doped fibers as new type of laser medium are attracting attention due to their unique optical properties, which provide a way for the development of efficient optical devices (lasers, optical amplifiers, etc.) operating in broad spectral region from 1.1 to 1.8 μm^1^. Excluding wavelengths near 1550 nm, this spectral region remains rather uncovered because of the absence of optical transitions in fibers doped with rare-earth ions and other dopants^2^. Although at ~1550 nm there exist a great variety of efficient fiber lasers and amplifiers based on erbium-doped fibers, the practical gain in these fibers is limited to a relatively narrow spectral window between 1530–1560 nm. Outside of this region, the efficiency of the devices is decreased significantly. To date there has been significant progress in the field of development of Bi-doped fibers which has led to fabrication of the fibers producing gain in a broad wavelength range. As a result, continuous-wave (CW) fiber lasers, optical amplifiers and superluminescent sources based on bismuth fibers operating in the wavelength range from 1.15 to 1.55 μm have been successfully developed (see e.g.^3^ and references therein). The efficiency and output power of this type of fiber lasers could reach higher than 60% and 20 W, respectively.

Recently it was shown that using of high-GeO_2_ silica-based fiber doped with bismuth the spectral range of amplification could be extended to long-wavelength region from 1.6 to 1.8 μm^4^. Thereafter, CW bismuth-doped fiber lasers operating in 1.625–1.775 μm and providing a watt-level power output have been demonstrated^5^. This spectral region is very attractive for many applications, including tomography^6^, gas sensing^7^ and remote control. Moreover, human tissues containing lipids show a peak of absorption at ~1720 nm, absorption by fat exceeding the absorption caused by water at this wavelength. This allows selective targeting of tissues containing lipids, and could be exploited for example in different skin treatments^8^.

In addition to CW bismuth-doped fiber lasers, mode-locked lasers operating at all major wavelengths covered by CW bismuth fiber lasers have been demonstrated. Most of the demonstrations have been based on passive mode-locking using semiconductor saturable absorber mirrors (SESAMs) as mode-lockers^9^,^10^,^11^,^12^. Moreover, mode-locking at 1180 nm using a carbon nanotube (CNT) absorber has been demonstrated in^13^, where the authors reported generation of 558 ps and 4.7 ps pulses in anomalous and normal dispersion regimes, respectively. Recently, we demonstrated a 1.45 μm pulsed bismuth fiber system operating in normal dispersion regime and producing 240 fs pulses, using carbon nanotubes as a saturable absorber^14^. In this paper, we report the first mode-locked bismuth-doped fiber laser operating at 1.7 μm, extending the mode-locked operation of bismuth fiber lasers to cover the long-wavelength gain region of bismuth-doped fibers. CNT saturable absorber has been used successfully to obtain pulse generation in both anomalous and normal cavity dispersion regimes.

## Results

**Figure 1:**
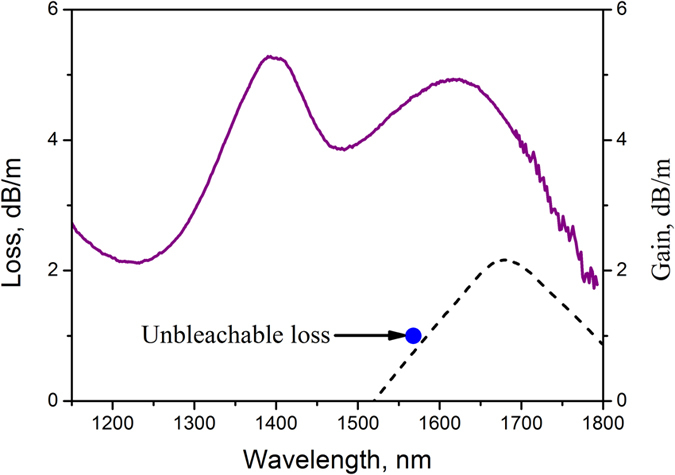
Absorption and gain spectra of bismuth-doped fiber are plotted by solid and dashed lines, respectively. Unbleachable loss level at 1.55 μm is indicated as a point.

Single-mode active medium was composed of a bismuth-doped fiber with an equal content of GeO_2_ and SiO_2_ in a core. The fiber preform was fabricated by a standard modified chemical vapor deposition (MCVD) process. The fiber with 125-μm diameter and cutoff wavelength of 1.2 μm was drawn from the preform after additional jacketing made at the same drawing speed. Bismuth concentration in the fiber core glass was ~0.02 wt.%. The absorption spectrum of bismuth-doped fiber with two characteristic bands is presented in Fig. 1. The absorption bands peaked at 1.4 and 1.65 μm caused by the formation of bismuth-related active centers are associated with Si and Ge, respectively. The optical properties of these bismuth-related active centers can be found in detail in^15^, where the study is focused on the optical properties of the bismuth-doped fiber primarily for a spectral range of 1.6–1.8 μm. First, the loss measurements were carried out at 1.56 μm as a function of pump power. The residual loss level shown in Fig. 1 is ~1 dB/m, corresponding to the small signal absorption of 22%. The high level of unbleachable loss is due to the relatively high concentration of bismuth ions required for laser with a short cavity length. Figure 1 illustrates the gain spectrum of bismuth-doped fiber pumped at 1.46 μm. The highest small-signal gain of ~2 dB/m was observed at the wavelength 1.7 μm for pump power of ~200 mW.

Two single-walled carbon nanotube (SWNT) saturable absorbers were prepared for the mode-locking experiments. The carbon nanotubes were produced by thermal composition of ferrocene in the presence of carbon monoxide at ambient pressure^16^. SWNTs were collected onto a nitrocellulose filter during the fabrication, so that a thin SWNT-film was formed onto the filter surface. The carbon nanotube film was then transferred onto a silver mirror by pressing the filter and SWNT-film (SWNT-film side down) against the mirror surface. The carbon nanotube film was strongly adhered on the mirror surface as a result of applied pressure, after which the remaining filter was peeled off leaving a pure SWNT-film on the mirror surface. The other saturable absorber mirror was fabricated by stacking two layers of SWNT-film onto a mirror, whereas the other absorber consisted of three layers of SWNT-film. As it is well known, major drawbacks of CNT-absorbers are their relatively high non-saturable losses and low ratio of modulation depth to the non-saturable loss^17^. Particularly in case of fiber lasers, a stable mode-locking needs sufficient value of modulation depth, whereas the fiber lasers can often tolerate considerable amount of non-saturable losses because of usually high values of gain. Therefore, multiple layers of carbon nanotube film were needed to increase the modulation depth of the absorber, although this unavoidably increased the non-saturable losses of the absorber as well. At least two layers of SWNT-film were found to be vital in order to achieve stable mode-locked operation and suppress the parasitic continuous wave oscillations, which were encountered when using an absorber with just one layer of SWNT-film.

**Figure 2:**
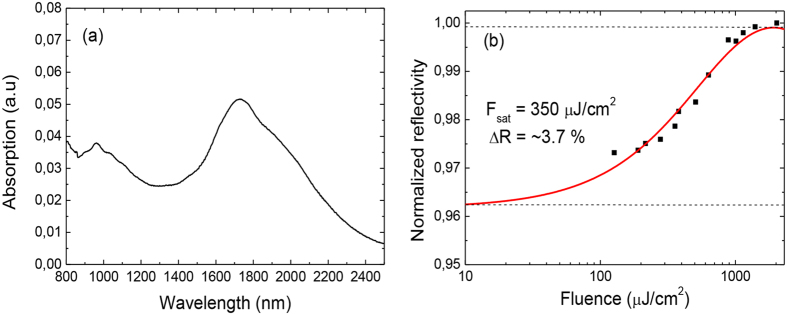
(**a**) The linear absorption of a single SWNT layer. The fabricated two saturable absorber mirrors exploited two and three layers of SWNT layers stacked one on another, increasing the both the linear and nonlinear absorption of the absorber. (**b**) The normalized nonlinear reflectivity of the absorber equipped with two layers of SWNT film.

The optical properties of carbon nanotubes are dependent on the diameter of the nanotubes^18^. The diameter of the used nanotubes ranged from about 1.2 nm to 1.8 nm, meaning that the nanotubes were slightly larger than often used for absorbers for pulsed lasers at ~1500 nm wavelength^19^,^20^. The linear absorption of a SWNT-film was measured by transferring a single layer of carbon nanotube film onto a quartz glass substrate, and the absorption spectrum was recorded by a spectrometer which covered the working wavelength range from 175 to 3300 nm. An uncoated substrate was used as a reference to exclude the effect of the substrate and light source. The linear absorption of a one SWNT-film is shown in Fig. 2(a). For used CNT-absorbers exploiting two/three layers of carbon nanotube film, the linear absorption is about two/three times the absorption of a one layer, respectively. This leads to linear absorption values of about 10% and 15% at 1700 nm for the two used absorbers.

The nonlinear optical properties were measured at 1700 nm wavelength by using pump-probe spectroscopy^21^. The normalized nonlinear reflectivity of the absorber equipped with two layers of CNT is presented in Fig. 2(b) showing the modulation depth of ~3.7%. The nonlinear reflectivity of the second absorber is estimated to be slightly higher. Compared for example to recent work in^19^ and^22^, the modulation depth of the absorber is lower compared to the previously reported value of ~11%. Moreover, the authors report saturation intensities considerably lower than that of the absorbers used in this work.

**Figure 3:**
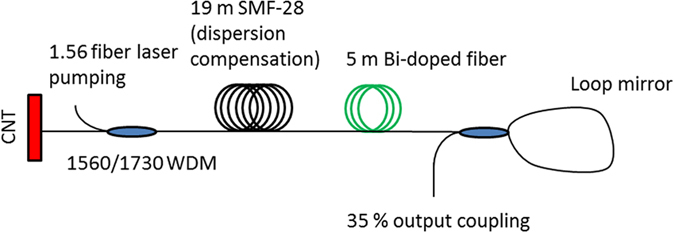
The schematic of the mode-locked bismuth fiber laser operating in the anomalous dispersion regime.

**Figure 4:**
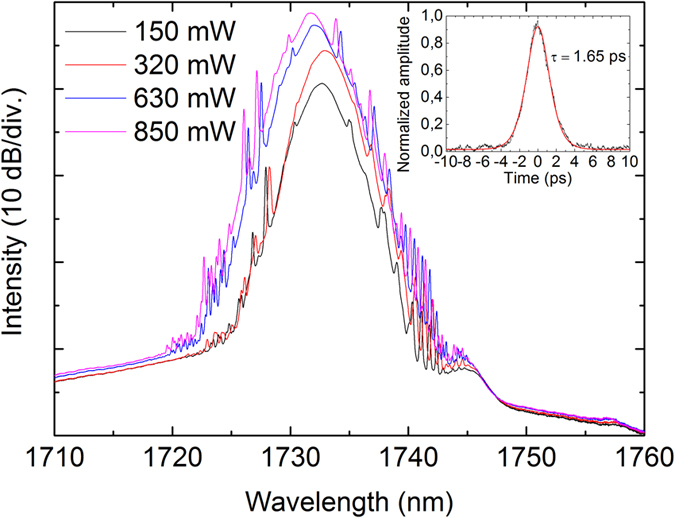
The spectra at different pump powers and the autocorrelation trace (inset) of the output pulse for the cavity operating in anomalous dispersion regime.

After the fabrication of fibers and preparation of saturable absorbers, a mode-locked bismuth fiber laser operating at 1.7 μm was studied for two dispersion regimes. The cavity is shown schematically in Fig. 3. In anomalous dispersion regime the cavity comprises 5 m long active fiber with normal dispersion and 19 meters of standard telecom fiber used for dispersion compensation. The dispersion management ensures the operation in net anomalous dispersion regime with a total cavity dispersion of ~0.35 ps/nm. The pump radiation at 1565 nm from continuous-wave erbium fiber laser is launched through a dichroic 1560/1730 nm pump coupler. One end of the cavity is terminated by a saturable absorber with two layers of CNT-film, while another cavity end is terminated by a fiber loop mirror directing 35% of the signal to the output. Residual pump is filtered out using two selective couplers placed at the output port of the laser. Above 300 mW of pumping power, a stable mode-locked operation of the laser has been regularly observed. The spectra and corresponding autocorrelation in anomalous-dispersion soliton regime are presented in Fig. 4.

**Figure 5:**
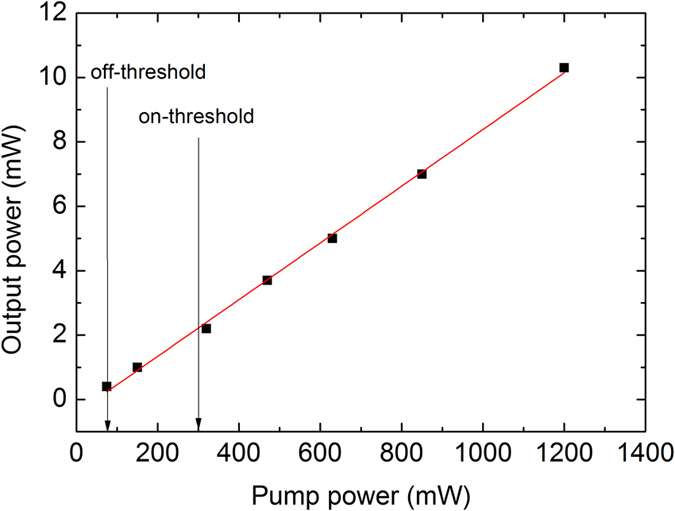
The average output power versus pump power. The pulsed operation develops when pump power is increased to 300 mW (“on-threshold”). When decreasing the pump power, the pulsed operation is maintained until the pump power is decreased below 75 mW (“off-threshold”).

**Figure 6:**
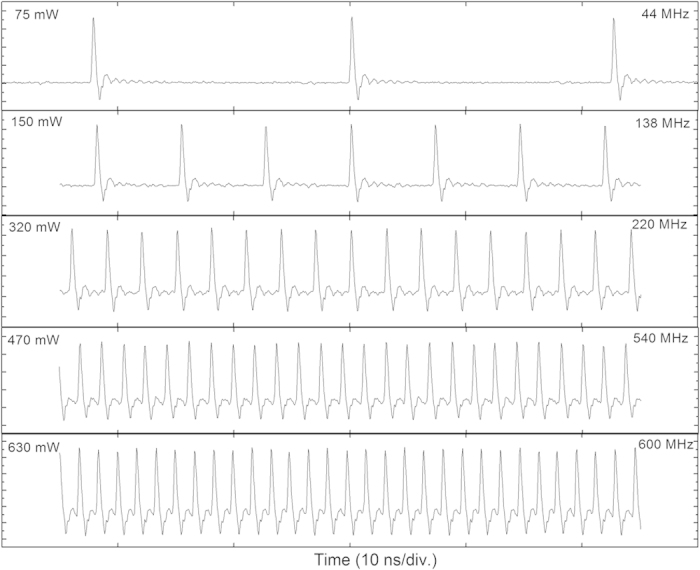
The pulse trains for different pump powers.

The pulse spectrum centered at 1730 nm has the bandwidth of 2.91 nm, as seen from Fig. 4. The laser produces 1.65 ps sech^2^-pulses with time-bandwidth product of 0.48 indicating nearly transform-limited pulse quality. The slight deviation from the transform limited quality is because of the chromatic dispersion in relatively long fiber pigtails at the laser output. With an increase of pump power, the laser demonstrates a strong tendency to multiple-pulse operation, which is a typical dynamics of a soliton laser operating with net anomalous dispersion cavity. The multiple pulse instability is a dominant mechanism limiting the pulse energy scaling in conservative soliton systems. For the start-up of the pulse operation corresponding to the pump power of 300 mW (“on-threshold” of mode-locking), the pulse repetition rate is ~200 MHz, though the fundamental repetition rate is only ~4 MHz. The laser exhibits a large hysteresis of pulse operation, since the mode-locking is switched off only after the pump power is decreased to below 75 mW, which is marked as “off-threshold” in Fig. 5. With increasing the pump power, the laser tends to produce stable harmonic mode-locked operation with well-defined pulse periods (Fig. 6), contrary to soliton bunching and grouping phenomena often observed in fiber lasers operating in anomalous dispersion regime. Regarding the stability of the pulse train, for many applications the property known as timing jitter is important, since it describes the deviations in temporal pulse positions compared to a perfectly regular pulse train. Timing jitter of the laser was not measured. However, we note that timing jitter is provoked by the cavity and pumping instabilities, and therefore in case of free-running passively mode-locked laser timing jitter can be relatively high compared to laser systems where special care has been taken to reduce the timing jitter.

The cavity dispersion was then changed to normal dispersion regime by reducing the length of additional intracavity anomalous dispersion fiber from 19 meters down to 10 meters, resulting in the net normal dispersion of −0.1 ps/nm. It was found that larger value of modulation depth and absoprtion was required to maintain stable pulse operation in normal dispersion regime. Thus, a mirror comprising three layers of CNT-film was used as a saturable absorber for the normal dispersion cavity, while the modulation depth of two layers was sufficient for the net anomalous dispersion cavity. We note that it is possible to use the same absorber for triggering mode-locking in both anomalous and normal dispersion regimes, as demonstrated for example in^23^. Indeed, it was observed that the absorber with higher nonlinearity was capable of triggering mode-locking in anomalous dispersion regime as well. However, the performance of the laser was optimized by using an absorber with lower absorption value in anomalous dispersion regime.

**Figure 7:**
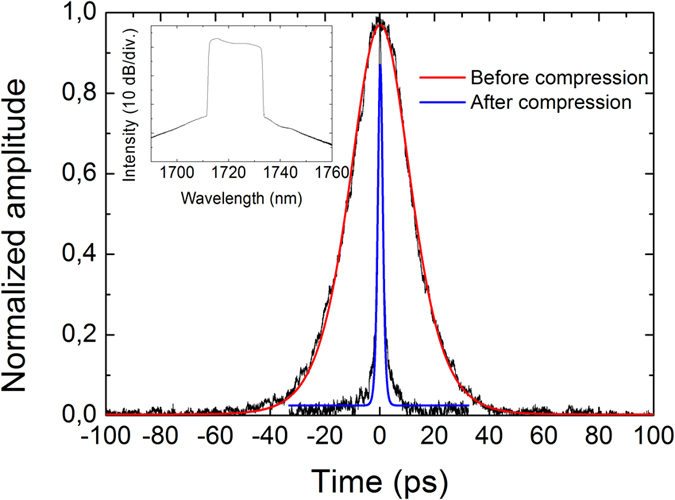
The autocorrelation traces of the pulse with normal cavity dispersion before the compression (red) and after the external pulse compression (blue). The spectrum is shown as an inset. The pump power is 170 mW.

**Figure 8:**
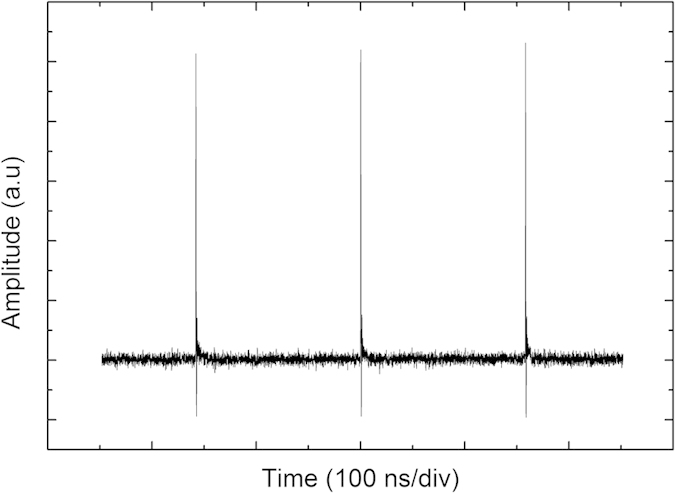
The single-pulse train from the normal dispersion cavity.

For the normal dispersion, a single pulse operation was observed after increasing the pump power over “on-threshold” of pulse regime. The pulse operation could be maintained until the pump power was decreased below the “off-threshold” value, showing hysteresis behavior comparable to the anomalous dispersion regime. The hysteresis phenomenon and multiple pulse formation in cavities with normal dispersion have been observed and are discussed more detailed for example in^24^. In our laser the threshold for possible multiple pulse operation was not reached in normal dispersion regime. Figures 7 and 8 present characteristics of the single pulse train, revealing 14-ps pulses with ~20 nm spectral bandwidth. The pulses are highly chirped as expected for a cavity with normal dispersion. With further increase of the pump power, the optical spectrum exhibits essential broadening and some instability of the pulse train was developed.

Finally, the compressibility of the pulse was studied by adding standard telecom fiber (SMF-28) to the output of the laser. Figure 7 shows the pulse characteristics before compression (red curve) and after propagation through 70 m of SMF-28 fiber (blue curve). The output pulse width of 1.2 ps assuming sech^2^-fitting indicates an efficient compression of the pulse. Further compression of the pulse would require optimization of the compressor and transform-limited pulse width of ~160 fs could be approached with optimized length of the fiber. However, in practice the shortest pulse duration is limited by the chirp nonlinearity.

## Conclusion

In conclusion, we have demonstrated a mode-locked bismuth doped fiber laser operating in 1.7 μm spectral range. Pulse operation has been demonstrated using carbon nanotube (CNT) as a broadband saturable absorber for the laser cavity with both anomalous and normal dispersion. In anomalous dispersion regime nearly transform limited pulses with pulse duration of 1.65 ps were achieved; however, the pulse energy was limited by the multiple pulsing developed with increasing pump power. In normal dispersion regime the laser operated in a stable single-pulse regime producing 14 ps pulses, which could be compressed down to 1.2 ps using an external fiber compressor. It can be therefore concluded that the mode-locked bismuth-doped fiber lasers are capable to operate at long-wavelengths of 1.75 μm, representing an attractive pulse source suitable for numerous applications.

## Additional Information

**How to cite this article**: Noronen, T. *et al.* 1700 nm dispersion managed mode-locked bismuth fiber laser. *Sci. Rep.* **6**, 24876; doi: 10.1038/srep24876 (2016).
